# Study Protocol for EXPRESS Fall Prevention Trial Among Older Adults in Rural China

**DOI:** 10.1093/geroni/igaf122.2642

**Published:** 2025-12-31

**Authors:** Shaojie Li, Yao Yao, Wenjian Zhou, Yuling Jiang, Mingzhi Yu, Yifei Wu, Jing Wu, Zhenhan Yu

**Affiliations:** Peking University, Beijing, Beijing, China (People’s Republic); Peking University, Beijing, Beijing, China (People’s Republic); Peking University, Beijing, Beijing, China (People’s Republic); Peking University, Beijing, Beijing, China (People’s Republic); Peking University, Beijing, Beijing, China (People’s Republic); Peking University, Beijing, Beijing, China (People’s Republic); Peking University, Beijing, Beijing, China (People’s Republic); Peking University, Beijing, Beijing, China (People’s Republic)

## Abstract

Falls constitute a leading cause of injury-related mortality and morbidity among older adults, especially in rural China where healthcare resources are limited. To address this, we designed the EXPRESS trial (EXpress Prevent Rural oldEr adults’ fallS, fractureS and dependency), a multicenter, community-based, cluster-randomized controlled trial integrating artificial intelligence (AI), village doctors, and Express Mail Service (EMS) providers. The study involves 16 villages randomized into intervention and control groups across four geographically diverse provinces in China. The intervention employs the innovative “XVV” strategy: a comprehensive ten-item fall risk screening by village doctors, AI-assisted smartphone-based gait analysis covering five biomechanical dimensions (velocity, stride variability, symmetry, stability, mobility), and five personalized, culturally adapted interventions delivered via village doctors and EMS couriers at key cultural moments (Chongyang, Spring Festival, Qingming Festival). The primary outcomes are the incidence of falls and related injuries over 12 months. Secondary outcomes include changes in quality of life (EQ-5D-5L), activities of daily living (ADL), and fall prevention literacy. Baseline data collection was completed in August 2024, with final evaluations scheduled for August 2025. A health economics evaluation will assess cost-effectiveness. By integrating AI, task-sharing, and community-based services, EXPRESS aims to demonstrate a scalable and sustainable model for fall prevention. Results will inform public health strategies globally, especially in low-resource, rural contexts, bridging the significant gap between fall prevention evidence and practice implementation. We hope to present a detailed plan for this study at the GSA conference.

